# Sacroiliac Joint Asymmetry Regarding Inflammation and Bone Turnover: Assessment by FDG and NaF PET/CT

**DOI:** 10.22038/AOJNMB.2019.40820.1275

**Published:** 2019

**Authors:** Abdullah Al-Zaghal, Dani P. Yellanki, Esha Kothekar, Thomas J. Werner, Poul F. Høilund-Carlsen, Abass Alavi

**Affiliations:** 1 Department of Radiology, Hospital of the University of Pennsylvania, PA, USA; 2 Department of Nuclear Medicine, Odense University Hospital, Odense, Denmark; 3 Institute of Clinical Research, University of Southern Denmark, Odense, Denmark

**Keywords:** FDG, Laterality, Leg-Dominance, NaF, Sacroiliac

## Abstract

**Objective(s):**

This study was undertaken to determine the role of computed tomography (CT)-based methodology to segment the SI joint and quantify the metabolic activity using positron emission tomography (PET). We measured tracer uptake in the right and left SI joints independently to look for differences between the two sides. Further, we correlated tracer uptake with BMI and studied the inter-observer variation with regard to estimated tracer uptake in the SI joints.

**Methods:**

In this retrospective study, a total of 103 subjects (48 females, 55 males) from the CAMONA study database collected 2012-2016 at Odense University Hospital in Denmark were included. Mean age was 48±14.59 years, mean BMI was 26.68±4.31 kg/m^2^. The SI joints were segmented on fused PET/CT images using a 3D growing algorithm with adjustable upper and lower Hounsfield Units (HU) thresholds. The metabolic activities on the two sides were correlated with BMI.

**Results:**

For FDG, we found a higher average SUV_mean_ on the right side (right: 1.3±0.33, left: 1.13±0.30; <0.0001). Similarly, for NaF, the uptake was higher on the right side (right: 5.9±1.29, left: 4.27±1.23; <0.0001). Positive correlations were present between BMI and FDG uptake (P<0.01) as well as NaF uptake (P<0.01).

**Conclusion:**

The PET-based molecular imaging probes along with the CT-based segmentation techniques revealed a significant difference in the metabolic activity between the two SI joints with higher inflammation and reactive bone formation on the right side. FDG and NaF uptakes correlated significantly and positively with BMI.

## Introduction

Sacrum is a large triangular bone at the base of the spinal column, formed by the fusion of vertebras S1-S5. It articulates with iliac bones on both sides to form the sacroiliac (SI) joint. The SI joint lies at an oblique angle to the sagittal plane, extending from the vertebral level of S1 to S3 (1). The SI joint has a narrow joint space that does not exceed 3 mm in its maximum width in young adults and decreases with aging (1, 2). Ligaments supporting the joint and maintaining its stability are found anteriorly and posteriorly in addition to the interosseous ligaments. The SI joint has a very limited range of motion about only 2-3 degrees, which is necessary for the joint to maintain stability and allowing for continuous microstructural adaptation to the superimposed biomechanical forces (3). 

Among its main functions, the SI joint transmits vertical force between upper torso and lower limbs, absorbs shocks generated by lower limbs, and helps in maintaining the upright spine posture against gravity (4). Age-related changes start in early adulthood and progresses with time. It begins with an increase in the articular surface roughness, followed by an increase in surface irregularity, subchondral bone sclerosis, and crevice formation, with a significant restriction of motion noted after the seventh and eight decades of life (5). Obesity has been associated with low back pain, as the increase in body weight increases the mechanical workload on the joint and contributes to joint degeneration (6). 

There has been a growing interest for the use of molecular imaging to evaluate benign musculoskeletal disorders (7, 8). ^18^F-fludeoxyglucose (FDG) has proven its high sensitivity in detecting inflammation in its earliest stages and made it possible to measure the metabolic activity using standardized uptake value (SUV). ^18^F-sodium fluoride (NaF) is a radiopharmaceutical tracer used to quantify calcium metabolism and bone turnover, and it is mainly used to evaluate skeletal involvement in malignant diseases. Recent studies have suggested the utilization of NaF in some benign skeletal disorders; like osteoarthritis, osteoporosis, and rheumatoid arthritis, and described its sensitivity in detecting the earliest metabolic changes of the disease before structural changes become evident with conventional imaging modalities (9).

 This study was undertaken to determine the role of computed tomography (CT)-based methodology to segment the SI joint and quantify the metabolic activity using positron emission tomography (PET). We measured tracer uptake in the right and left SI joints independently to look for differences between the two sides. Further, we correlated tracer uptake with BMI and studied the inter-observer variation with regard to estimated tracer uptake in the SI joints.

## Methods

FDG and NaF PET/CT scans utilized in this retrospective study are part of the “Cardiovascular Molecular Calcification Assessed by ^18^F-NaF PET/CT” (CAMONA) protocol. CAMONA was approved by the Danish National Committee on Health Research Ethics, registered at ClinicalTrials.gov (NCT01724749), and conducted from 2012 to 2016 in accordance with the Declaration of Helsinki. Written informed consent was obtained from all study subjects. Detailed description of the CAMONA study was previously published by Blomberg BA, et al. (10)

***Subject Selection***

The CAMONA study consists of 139 study subjects; 89 healthy control subjects, and 50 patients with a history of chest pain. Healthy volunteers were recruited from the general population by local advertisement or from the blood bank of Odense University Hospital, Denmark. Subjects with a negative history of cardiovascular disease, oncologic disease, autoimmune disease, immunodeficiency syndromes, alcohol abuse, illicit drug use, or any prescription medication were considered as healthy volunteers. Framingham Risk Score (FRS) was used to evaluate the modifiable cardiovascular risk factors and only subjects with score below the upper limits of the recommended levels (i.e. blood pressure below 160 mm Hg systolic and 100 mm Hg diastolic, total serum cholesterol below 6.2 mmol/L, glycated hemoglobin (HbA1c) below 48 mmol/L, and a non-smoking status) were included. Pregnant women were not included within the study population. Detailed description of the healthy subjects included in the CAMONA study were previously published by Blomberg BA, et al.(11).

Subjects with history of chest pain were recruited among patients referred to the cardiology department for a coronary CT-angiography. Only patients with a 10-year risk for fatal cardiovascular disease equal to or above 1%, as calculated by the body mass index (kg/m^2^) based Systematic Coronary Risk Evaluation (SCORE) tool, were eligible for inclusion. Subjects with a history of major cardiovascular events, malignancy, chronic inflammatory disease, illicit drug use, renal insufficiency were not included. Further details regarding the inclusion criteria for the unhealthy subjects of the CAMONA study were previously published by Blomberg BA et al(10). 

Twenty-seven subjects were excluded, as either their NaF or FDG scans were not available in our lab’s database. Seven subjects were excluded as their scans had technical issues prevented image analyses. Two subjects were excluded, as the desired region of interest (pelvis) was not present within the field of imaging. A total of 103 subjects, 55 males and 48 females who had both their FDG and NaF PET/CT scans with the desired regions of interest (pelvis) within the field of imaging. Mean age was 48±14.59 years with a range of 21-75 years. Mean BMI was 26.68±4.31 kg/m^2^.

***Study Design***

Information regarding subjects smoking habits, family history of CVD, and prescription medication were acquired through questionnaires. Fasting serum total cholesterol, serum low-density lipoprotein (LDL) cholesterol, serum high-density lipoprotein (HDL) cholesterol, fasting plasma glucose and glycated hemoglobin (HbA1c) were measured. The estimated glomerular filtration rate (eGFR) was calculated using the Modification of Diet and Renal Disease (MDRD) equation. Three blood pressure (BP) measurements were acquired after the subject had rested for of at least 30 min in the supine position. The average of the last two measurements was recorded as the systolic and diastolic blood pressure. In each subject, the 10-year risk of developing CVD was approximated using the Framingham risk score (FRS) (i.e. risk of coronary death, myocardial infarction, coronary insufficiency, angina, ischemic stroke, hemorrhagic stroke, transient ischemic attack, peripheral artery disease, heart failure) based on age, gender, systolic blood pressure, total serum cholesterol, serum HDL cholesterol, smoking habit, and management for hypertension (12).

The protocol of FDG and NaF PET/CT imaging was previously published by Blomberg BA, et al (13, 14). In summary, ^18^F-FDG and Na^18^F PET/CT imaging were performed on hybrid PET/CT systems (GE Discovery STE, VCT, RX, and 690/710 systems). Each subject was randomly allocated to the PET/CT system by the department’s booking system. ^18^F-FDG PET/CT imaging was performed 180 minutes after intravenous administration of 4.0 MBq of ^18^F-FDG per kilogram of body weight. ^18^F-FDG was injected after an overnight fast of at least 8 hours and a confirmed blood glucose concentration of below 8 mmol/L. On average, ^18^F-Na PET/CT imaging was performed within 14 days of ^18^F-FDG PET/CT imaging. ^18^F-NaF PET/CT imaging was performed 90 min after intravenous administration of 2.2 MBq of ^18^F-Na per kilogram of body weight. PET images were corrected for scatter, attenuation, random coincidences, and scanner dead time. Low–dose CT imaging (140 kV, 30–110 mA, noise index 25, 0.8 second/rotation, slice thickness 3.75 mm) was performed for attenuation correction and anatomical orientation. The effective radiation dose received from the entire imaging protocol was approximately 14 mSv.

***Image Analyses***

**Figure 1:**
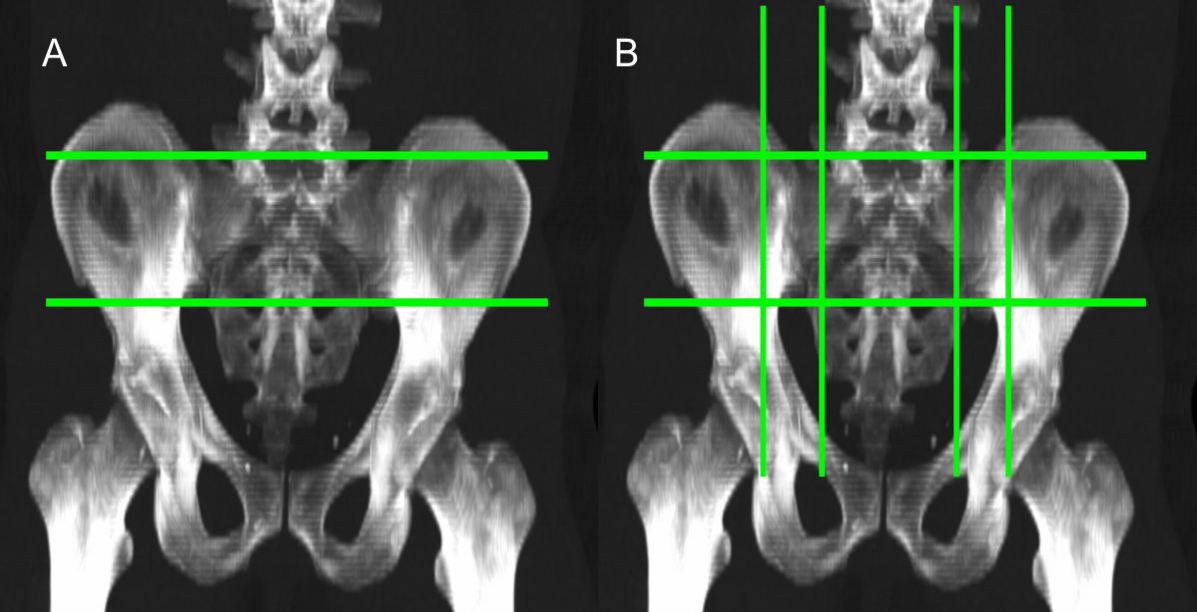
3D MIP images showing methodology to acquire a desired section of the SI joint using scissors editing tool. (A) Two lines parallel to the axial axis were drawn at the upper and lower ends of the joint space. (B) Two lines parallel to the sagittal axis marked the medial and lateral borders of the SI joint; the medial border went through the midpoint between anterior sacral foramina and joint space, while the lateral border was around 2 cm lateral to joint space

OsirX MD v.9.0 (DICOM viewer and image-analysis program, Pixmeo SARL; Bernex, Switzerland) was used for image analysis. Regions of interest (ROIs) were assigned according to predetermined anatomical criteria on 3D maximum intensity projection (MIP) images. Scissors editing tool was used to acquire the ROI of the SI joint on coronal view (Figure 1). Two lines parallel to the axial axis were drawn at the upper and lower ends of the joint space. Two lines parallel to the sagittal axis marked the medial and lateral borders of the SI joint; the medial border went through the midpoint between anterior sacral foramina and joint space, while the lateral border was around 2 cm lateral to joint space Then on axial view, an oblique line was drawn to exclude the posterior-lateral part of the iliac bone. The high variability of the pelvic anatomy among subjects was taken into consideration.

**Figure 2:**
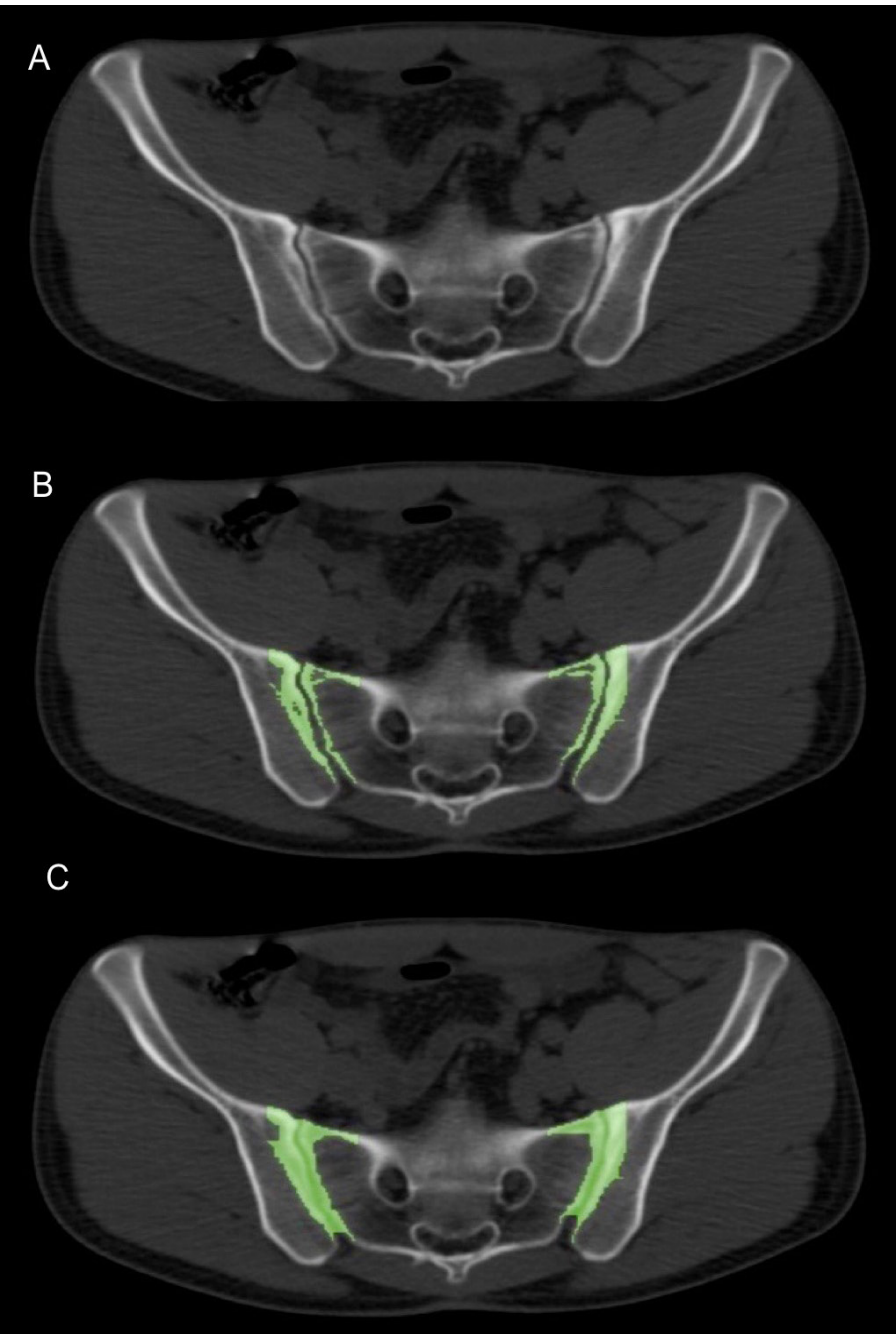
Using a 3D growing algorithm on the axial view (A), a Hounsfield unit threshold of 200 was assigned (B), followed by a closing algorithm with a structuring element radius of 4±2 to include subchondral bone and joint space respectively

A 3D growing region algorithm with a lower Hounsfield Unit (HU) threshold of 200 was assigned on fused PET/CT images, to include subchondral bone. It was followed by a morphological closing algorithm with a structuring element radius of 4±2 to include the joint space. Figure 2 shows the process of highlighting the ROI on axial PET/CT images. Total ROI SUV and pixels were measured for each axial slice and exported to a CSV file by Osirix software. 

The metabolic activity of each slice was calculated by multiplying the slice SUV_mean_ by the slice ROI_volume_. The total metabolism of all slices was summed up to measure the total metabolic activity of the investigated tissue compartment, as in the following equation:

Total Tissue metabolism=∑(Slice SUVmean*Slice ROIvolume

Averaged SUV_mean_ was used for the quantification of FDG and NaF according to the following equation: 

Averaged SUVmean=Total Tissue MetbolismROItotal_volume

**Figure 3:**
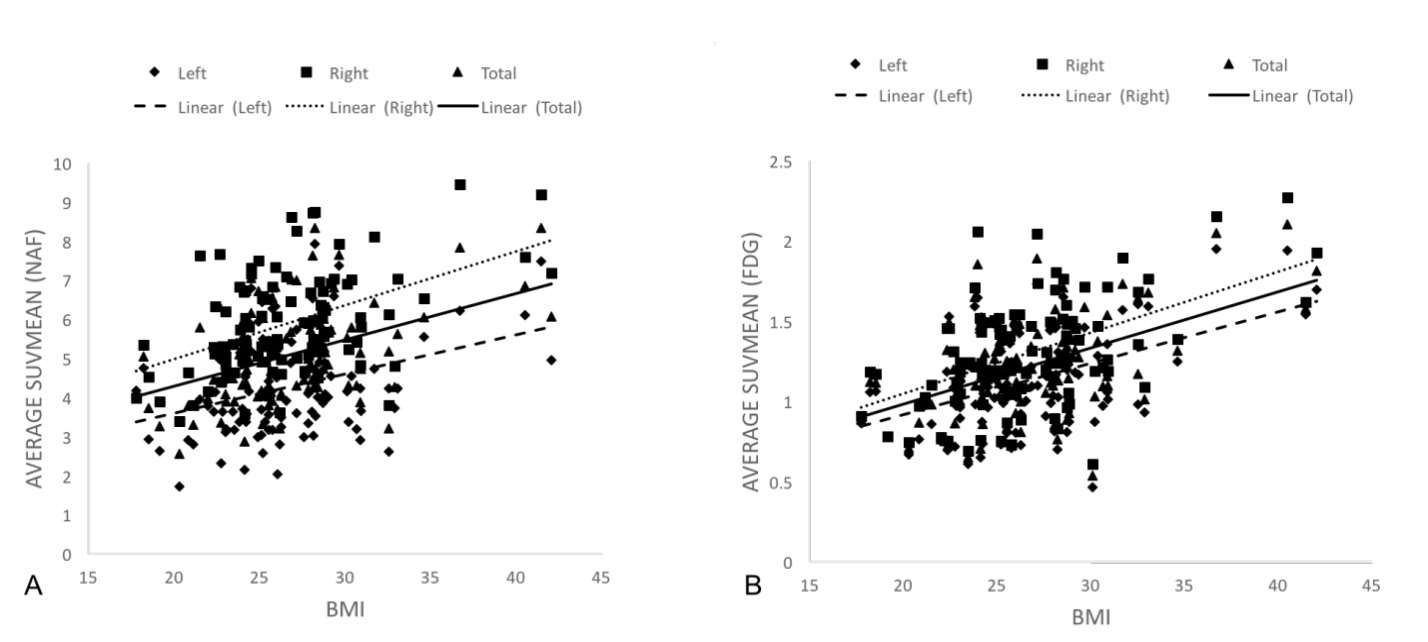
BMI correlating positively with both NaF (A) and FDG (B) uptake indicating increased bone turnover and inflammatory activity in the SI joint respectively

**Figure 4.**
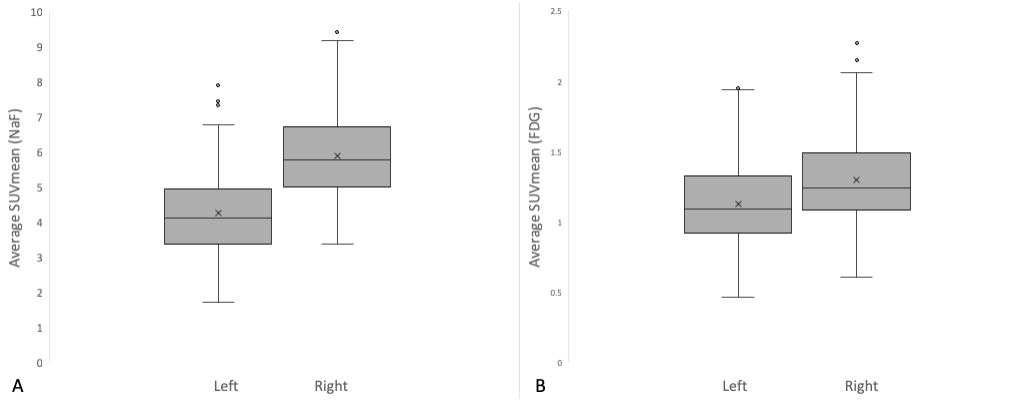
Right side has significantly higher tracer uptake for both NaF and FDG compared to the left side and could be attributed to right leg dominance

***Statistical analysis***

A two-tailed paired t-test was performed to evaluate the difference in tracer uptake on sides of the SI joint. Correlation analysis was conducted to assess the effects of BMI on tracer uptake in each side. A two-tailed P value below 0.05 was regarded statistically significant. Statistical analysis was performed by IBM SPSS Statistics v.25.0 (IBM Corp. Released 2017. IBM SPSS Statistics for Macintosh, Version 25.0. Armonk, NY: IBM Corp).

## Results

Mean FDG uptake was 1.29 (SD=0.32) on the right side while 1.09 (SD=0.28) on the left side (P<0.0001). Mean NaF uptake was 5.78 (SD=1.23) on the right side, while 4.12 (SD=1.20) on the left side (P<0.0001). A significant positive correlation was found between increasing BMI values and FDG uptake (right side: R=0.46, P<0.0001, left side: R=0.39, P<0.0001). A significant positive correlation was also found between increasing BMI and NaF uptake (right side: R=0.43, P<0.0001, left side: R=0.37, P<0.0001). Graphical representation of the correlation analysis is illustrated in Figures 3 and 4.

## Discussion

This scientific article describes an approach to segment the SI joint on PET/CT, allowing for the quantification of inflammatory activity and reactive bone formation as measured by FDG and NaF, respectively. 

The metabolic activity of the SI joint had a significant positive correlation with BMI for both FDG and NaF.

Body locomotion is a complex process initiated and controlled by the central nervous system and executed by lower limbs in coordination with the peripheral nerves (16). Axial forces generated by the upper body and lower limbs are transmitted caudally and dorsally, respectively, through the sacroiliac joint (15).

The symmetry of the lower limbs regarding motor function has always been an interesting field for researchers. Studies done in the past described normal gait in healthy people to be symmetric, where both lower limbs function in the same manner and identically contribute to moving the body forward and maintaining stable posture (16). Methods used at that time could not find any significant functional, mechanical or anatomical differences between the limbs. Seeley MK et al. investigated normal gait in 20 subjects and could not detect any significant functional asymmetry (17). 

Researchers opposing the concept of gait symmetry proposed the theory of lower limb dominance; in the normal bipedal gait, humans use one leg to initiate movement and push the body forward (dominant leg) while the contralateral limb stabilizes the body and trunk posture meanwhile (non-dominant leg) (18). A preference to use the right foot rather than the left in initiating walking, jumping or kicking a ball has been documented in the literature (19). A study done by Carey DP et al. included 236 soccer players participating in 1998 World Cup, concluded that 79% of players were right-footed (20). Lower limb lateralization has also been described in both running and cycling (21).

Increasing BMI is also associated with an increase in the body loads imposed on the SI joint and the mechanical force transmitted across the joint, consequently reflected as an increase in joint stress and inflammation (22). Increasing compressive forces on subchondral bone was associated with an increase in the expression of IL-6, COX-2 and IL-8 (23). Chondrocytes have mechanoreceptors that sense changes in the exerted mechanical stress and translate it into biochemical signals, which consequently regulate the production of pro-inflammatory mediators (22, 24). The expression of these pro-inflammatory mediators is significantly increased in the joints of overweight and obese subjects (25), keeping the joint in an active low-grade state of inflammation. The impact of body weight was higher on the right side as the association was stronger for both FDG and NaF uptake (26).

Bone is a dynamic tissue that keeps changing its micro-architecture in response to external influencing factors. The increase in mechanical loading increases the proliferation and differentiation of osteoblasts and osteocytes (27); promoting new bone formation. However, pro-inflammatory mediators and adipokines secreted into systemic circulation were found to induce osteoclast differentiation, promoting bone resorption (28). Thus, the skeletal system in obese subjects has an increase in both bone formation and resorption (26). 

NaF exchanges ^18^F with OH^-^ from the surface of hydroxyl-apatite (Ca_10_[PO_4_]_6_[OH]_2_); which is the major component of bone matrix(29). This reaction depends on the availability of binding site on surface area for ^18^F and amount of received blood flow (30). Bone mineral density is known to decrease in females passing the age of menopause, due to hormonal changes resulting in a decline in estrogen levels (31). The decrease in bone density might affect the amount of hydroxyl-apatite leading to a decrease in the exposed surface area, consequently reducing the number of the binding sites available to react with NaF. 

The data generated in this study revealed a significant difference in the metabolic activity on the sides of the SI joint with a trend of higher uptake in the right side with 92.2 % (95/103) of FDG and 98% (101/103) of NaF uptake. The difference in tracer uptake might be attributed an underlying functional asymmetry in the joint, unequal weight distribution, pelvic or spinal pathologies or other miscellaneous etiologies.

The current study was limited by the shortage of clinical information regarding subjects’ history of any musculoskeletal disorders, as sacroiliitis, scoliosis, ankyloses or malalignment. It also would have been of a great value if we had knowledge of subjects’ hand or foot preference and correlated it with our quantitative findings.

## Conclusion

The CT-based segmentation techniques revealed a significant difference in the metabolic activity on sides of the SI joint with higher inflammation and reactive bone formation on the right side. It also showed a positive correlation with BMI and tracer uptake consistent with the published literature indicating an association between high BMI and impact on joints. Further studies with larger number of subjects are warranted to examine in more detail the association between inflammation and bone turnover, biomechanical factors, and clinical findings to explain the significance of the observed sacroiliac joint asymmetry.
